# Evaluation of the Antiparasitic, Antihepatotoxicity, and Antioxidant Efficacy of Quercetin and Chitosan, Either Alone or in Combination, against Infection Induced by *Giardia lamblia* in Male Rats

**DOI:** 10.3390/life13122316

**Published:** 2023-12-10

**Authors:** Bander Albogami

**Affiliations:** Biology Department, College of Sciences, Taif University, P.O. Box 11099, Taif 21944, Saudi Arabia; b.boqami@tu.edu.sa

**Keywords:** *Giardia lamblia*, giardiasis, Quercetin, Chitosan, antioxidant, oxidative stress, hepatotoxicity

## Abstract

*Giardia lamblia* (*G. lamblia*) is one of the most common protozoal infections and a key cause of malabsorption, some cases of mental developmental issues in children, and reduced body weight. The known antiparasitic medications, which are the standard drugs used for parasitic treatment, have several side effects and sometimes exhibit low efficacy. Therefore, the current study aimed to evaluate the treatment with quercetin (QC) or chitosan (CH), either alone or in combination, as possible alternative therapeutic agents that may alleviate the side effects of *G. lamblia* infections and restore the normal architecture of the intestinal muscles. They are investigated as alternatives to other routinely administered drugs that may gradually lose their efficacy due to human resistance to therapeutic agents. This study was carried out on 50 male albino rats that were divided into five groups with 10 rats in each group: the control group (Group I), the infected non-treated group (Group II), the infected group treated with QC (Group III), the infected treated group with CH (Group IV), and the infected group treated with a combination of QC and CH (Group V). The effect was first evaluated by counting the *G. lamblia* fecal cysts in the stool, examining histopathological sections of the intestine with the appearance of trophozoites in the infected group, and conducting a transmission electron microscopic examination of the tissues of the small intestine. Alterations in the biochemical parameters of liver and kidney function and the antioxidant enzymes in the liver tissues of SOD, CAT, and GSH, and non-enzymatic markers of lipid peroxidation (MDA) were evaluated. The results showed a significant decline in the number of parasites in the stool samples, with a marked elevation in the number of trophozoites in the intestinal sections of the infected non-treated group as compared to the infected treated groups. The last group, which was treated with a combination of QC and CH, showed the best results in terms of a decline in the infection rate of *G. lamblia* in stool samples, with a marked and clear improvement in the intestinal mucosa, regular muscles with normal enteric ganglions, and reduced rates of intestinal injuries caused by *G. lamblia* trophozoites. Both QC and CH had non-toxic effects on the biochemical parameters of the liver and kidneys, as well as pronounced antioxidant activities due to the elevation of SOD, CAT, and GSH in conjunction with a decline in the levels of MDA. A combination of QC and CH can be considered a potent antiparasitic, anti-hepatotoxic, and antioxidant therapeutic agent; it could constitute a promising alternative treatment agent against *G. lamblia* infection.

## 1. Introduction

Flagellated protozoa, known as *Giardia lamblia* (*G. lamblia*), is a unicellular binucleate parasite that is mostly found in water samples; worldwide, it is the leading cause of diarrheal disease [1]. *G. lamblia* causes giardiasis, which, according to the most recent statistics, affects about 30% of children in the Middle East [2].

Based on research conducted by Ankarklev et al. [3], more than 280 million people are infected with *G. lamblia* every year. Meanwhile, Pecková et al. [4] demonstrated that *G. lamblia* causes infections in about 200 million people per year, revealing that it infects a huge number of animal hosts. It lives in the intestine and jejunum and then facilitates the transmission of fecal parasites. Therefore, *G. lamblia* is considered the leading cause of both acute and chronic diarrhea and may be a cause of malabsorption and malnutrition in low-income countries, with a smaller percentage of cases in developed countries.

According to a report produced by Torgerson et al. [5], giardiasis causes about 200 million gastrointestinal diseases. Meanwhile, other studies reported that the main causes of giardiasis are the fecal contamination of the water supply or direct person-to-person contact in daycare centers.

Savioli et al. [6] noted that the WHO (World Health Organization) included *G. lamblia*-induced giardiasis in the Neglected Disease Initiative (NDI) in 2004, also mentioning the lack of information about the molecular mechanisms of this disease. Giardiasis generally manifests as weight loss, watery diarrhea, malabsorption, and malnutrition, and it is mostly detected in infants and children [7].

Common anti-parasitic therapeutics, such as albendazole or nitazoxanide, are considered alternative therapeutic agents, but in some previous studies, these agents appeared to be mutagenic, with some carcinogenic activity [8]. Additionally, it is well known that some major medications have significant side effects [9], and other medications appear to have a pronounced potential for resistance [10].

As a result of the findings of previous studies, it is necessary to conduct studies on herbal or active compounds with medicinal properties that might function as alternatives to common antiparasitic medications, especially against giardiasis. Natural active compounds are effective, exhibiting fewer side effects and, in many cases, demonstrating a therapeutic effect [11].

Quercetin (QC) is a common flavonoid found in many vegetables, fruits, and cereals. QC is the aglycone form of flavonoid glycosides, and it is mainly found in different food products, such as vinegar, black tea, and onions [12]. QC exhibits antioxidant properties against a range of diseases, including liver fibrosis, atherosclerosis, renal injury, and heart diseases [13].

Quercetin (QC) is a natural flavonoid that exhibits a lot of beneficial effects, such as anti-inflammatory, anti-diabetic, and anti-antioxidant capacities [14]. In addition, the administration of QC minimizes a number of oxidative stress injuries [15]. QC supplementation plays important roles in hepatoprotection, improves antioxidant activities, and minimizes oxidative damage [16].

Oxygen-free radicals are reactive species (ROS); in most cases, free radicals are generated in cellular living organs as a result of the regular metabolism. The excessive production of reactive free radicals has many deleterious effects on cells and can exert toxic effects on the cells of different organs [17].

Chitosan (CH) has attracted a significant amount of interest and attention due to its many biomedical activities. It exhibits several biological activities as an antitumor agent with antioxidant abilities and free-radical-scavenging activities [17].

Chitosan (CH) is composed of different sorts of active compounds, such as “glucosamine”, which are linked by glycosidic bonds [18]. Additionally, CH has been shown to enhance cell membrane permeability [19].

This study was designed to investigate the therapeutic, hepatoprotective, and antioxidant effects of QC and CH, either alone or in combination, against experimental giardiasis in male rats infected with *G. lamblia*; the study includes an assessment of their potential antioxidant activities.

## 2. Materials and Methods

### 2.1. Preparation of G. lamblia Cysts Used for the Inoculum of Infection

*Cysts of G. lamblia* were separated from the heavily diarrheic children stool samples. These six infected stool samples were collected in sterile, clean cups. Cysts of *G. lamblia* in routine saline smear were used to prepare the inoculum for infection. The samples with *G. lamblia* cysts were centrifuged for 5 min at 3000 r.p.m 3 times. The *G. lamblia* cyst count was conducted in 1 mL of stool sediment; the required infecting dose was calculated as 100,000 cysts/mL after averaging the three counts [20].

### 2.2. Ethical Statement

The current study was carried out following the ethical guidelines and animal care protocols; the current research proposal was approved by the ethical approval committee of Zagazig University under approval number ZU-IACUC/1/F/103/2023.

### 2.3. Determination of the Sample Size

The sample size of the current study was calculated by using the OPEN EPI software package, version 2.3.1, Assuming that CAT in hepatic tissue homogenates in the infected group treated with QC versus the infected group treated with both QC and CH (U\g), at a G-power of 80% and the confidence level of 95%, which recommended the use of ten rats/each group, thus the sample size is 50 (10/each group). 

**Figure 1:**
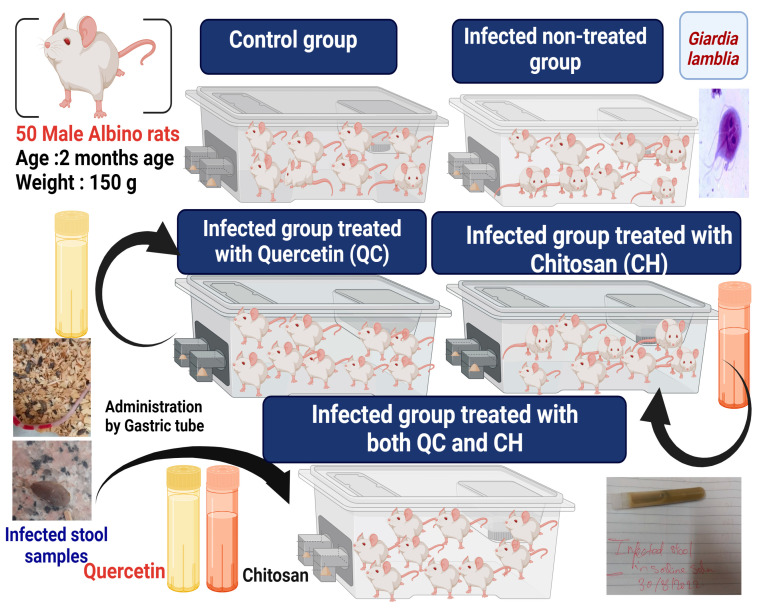
Experimental design of different treated groups.

The 50 male rats were divided into 5 groups. Group I: healthy control group (negative control); Group II: infected non-treated group; Groups III, IV, and V: infected groups, and then treated with QC, CH, and a combination of QC and CH, respectively (Figure 1). Each rat in Groups II–V was infected orally with *G. lamblia* cysts.

### 2.4. Compounds Used

Quercetin (QC) and Chitosan (CH) were obtained from Sigma Aldrich, Sigma, St. Louis, MO, USA, and were of a high purity and analytical grade. The two compounds were dissolved in a normal saline solution as a vehicle. QC was administrated orally in a dose of 30 mg/Kg [14]; meanwhile, CH was administrated orally in a dose of 1.5 g/Kg [19].

### 2.5. Experimental Animals

This experimental study was performed on 50 male albino rats of 2 months of age weighing 150 g. The male rats (All the animals were initially examined daily to ensure the absence of any of the parasitic infections) were obtained from the Theodore Bilharz Research Institute, Giza, Egypt, and were kept under suitable laboratory conditions with standard food and water *ad libitum*. The male rats were divided into five main groups, with 10 rats/each group, as shown in Figure 1. All treated groups were infected with *G. lamblia* except for the negative control group, which comprised healthy male rats. The other groups were infected with inoculation of a *G. lamblia* cyst suspension, containing nearly 100,000 cysts for each rat.

In this work, 50 male albino rats were divided into 5 groups of 10 male albino rats per group. Group I, the control group (-ve control), received normal physiological saline (0.9 N NaCl (1 mL/Kg)). Group II, the infected non-treated group (infected with the oral administration of *G. lamblia* ), did not receive any treatment. Group III was infected group with *G. lamblia* and then treated with QC (30 mg/Kg), Group IV was firstly infected with *G. lamblia* and then concurrently treated with CH (1.5 g/Kg), and Group V was infected with *G. lamblia* and then treated with a combination of QC and CH.

### 2.6. Assessment of the Efficacy of the Treatment

#### 2.6.1. Parasitological Evaluation

Fecal evaluations were performed for each rat using a normal saline Lugol’s iodine smear seven days after infection for three days to confirm the infection of the rats. To count the *G. lamblia* cysts after the last treatment dosage, 1 g of the stool samples was collected on three consecutive days using a hemocytometer to determine the mean number of cysts/HPF in the stained smears [21].

#### 2.6.2. Histopathological Evaluation

Cross sections of the small intestines of each rat were fixed in 10% neutral buffered formalin; then, normal procedures were used to make paraffin blocks and conduct sectioning to obtain slides with staining, using hematoxylin and eosin (H&E) as described by Ammar et al. [22], Examinations of the sections with a thickness of 4 µm were conducted microscopically, using both low and high power in the microscopic imaging unit at the Deanship of Scientific Research of Taif University, to assess and detect the degree of intestinal mucosal damage or recovery or the restoration of the normal state.

#### 2.6.3. Transmission Electron Microscope Examination (TEM)

Specimens of small intestines were taken from different treated groups. The obtained tissues were preserved after sacrificing the rats in glutaraldehyde (2.5%) for about four hours. The obtained specimens were excessively washed in “phosphate buffer solution” (pH~7.4) approximately 4 times/20 min and then suddenly post-fixed in 2% “osmium tetraoxide” and buffer solutions for 2 h. The fixed specimens were suddenly dehydrated in ascending grades of ethyl alcohol (30, 50, 70, 90, and 100%), then immediately cleared in “propylene oxide”, and then suddenly embedded in resin. The semi-thin sections were deeply stained with toluidine blue and then examined by using a light microscope. The desired sections were then stained with uranyl acetate and lead citrate for preparation of Ultrathin sections (60–90 nm thick) and imaged using a JOEL TEM unit (JEOL JEM-1200 EX II, Japan) and operated at 60–70 kV, according to the method described by Hayat [23].

#### 2.6.4. Assessment of Hepatorenal Functions

Blood samples were collected from the sacrificed male rats from the different infected and non-infected treated groups after they were subjected to light anesthesia with ketamine/xylazine. The samples were collected from the eye plexus, as this is the area that contains the most purified blood and bleeds most heavily. Serum samples were used for the determination of both liver enzymes (AST and ALT) and kidney functions (urea and creatinine); the analyses used commercial kits (Biodiagnostic Co., Birmingham, UK).

#### 2.6.5. Evaluation of Antioxidant Enzymes in Liver Tissues

Liver tissue homogenates were immediately prepared using cold buffer saline, and then we conducted an estimation of antioxidant enzymes and lipid marker peroxidation. Malondialdehyde (MDA) levels were estimated according to Ohkawa et al. [24]. Superoxide dismutase (SOD) activity was estimated based on Marklund and Marklund [25], catalase (CAT) was estimated according to Aebi [26], and glutathione levels (GSH) were evaluated according to Couri and Abdel-Rahman [27].

### 2.7. Statistical Analysis

Data were analyzed using the statistical analysis software package for the Social Sciences (SPSS) version 26 (IBM Corp., Armonk, NY, USA) (mean ± S.E). Comparisons between groups were made using two-way analysis of variance (ANOVA) with multiple post hoc tests, including the Duncan test. *p* values < 0.05 were considered statically significant [28].

## 3. Results

### 3.1. Parasitological Assessment of Infected Non-Treated and Treated Groups

#### *G. lamblia* Cyst Count in the Stools

**Figure 2:**
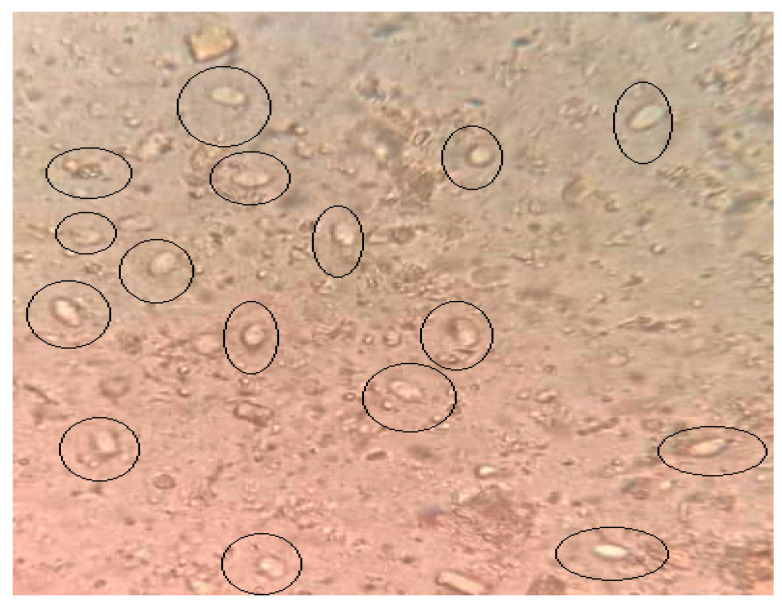
The presence of cysts of *G. lamblia* (black circles) in the iodine–saline-stained smear of heavily infected non-treated stool samples (400× magnification).

The cyst counts of *G. lamblia* in the stool samples revealed a significant reduction in the infected treated groups (Groups III, IV, and V) as compared to the infected non-treated group (Group II) (Figure 2). Additionally, a significant difference (*p* < 0.001) was noticed in Group V, which was treated with the combination of QC and CH, and the non-treated infected group. The results detected a reduction in the percentage of *G. lamblia* parasites. The results showed a non-significant difference between the infected groups treated with either QC or CH, with high and moderate reduction rates, 90% and 85%, respectively, as illustrated in Table 1.

### 3.2. Histopathological Examination Results

**Figure 3:**
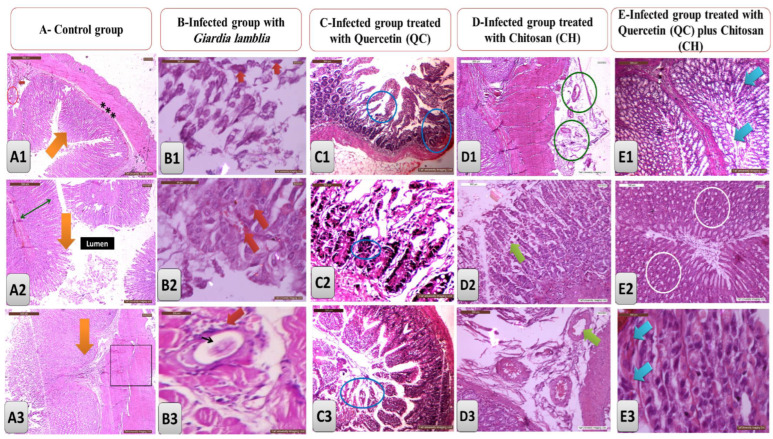
(**A1**–**A3**) The histological sections of the intestines of the control group showed normal sections with normal intestinal villi (orange arrows), normal serrated border of the intestinal villi and normal mucosa (double-headed green arrow), muscularis mucosa (***) and lamina propria (small red arrow), normal sized muscularis externa (Large square) and normal intestinal gland (Red circle) (H&E ×200). (**B1**–**B3**) histological sections of the intestines of infected rats with *Giardia lamblia* parasites with the appearance of trophozoites, then it became encysted (red arrow) and surrounded by fibrosis and inflammatory reaction, and replacement of the parasite with eosinophilic exudates (Black arrow) (H&E ×200). (**C1**–**C3**) histological sections of the intestines of infected rats with *Giardia lamblia* and then treated with quercetin (QC) showing inflammatory infiltrates (blue circles) in the intestinal crypts with normal appearance of intestinal glands and absence of the parasite (H&E ×200). (**D1**–**D3**) histological sections of the intestines of infected rats with *Giardia lamblia* and then treated with chitosan (CH), showing a high recovery rate (green arrow) with complete absence of the *Giardia lamblia* and any residue in the cysts present (green circles) (H&E ×200). (**E1**–**E3**) histological sections of the intestines of infected rats with *Giardia lamblia* and then treated with a combination of quercetin (QC) and chitosan (CH), showing very high rates of recovery with normal intestinal appearance (white circles), normal intestinal glands with absence of any shape of infection except for only very small amount of inflammatory cells as remnants (blue arrow) (**E1**,**E2**) (H&E ×200), and (**E3**) (H&EX400).

A histopathological examination of the sections of the small intestines from the normal control group (-ve control group) revealed normal intestinal mucosal layers with intact goblet cells (Figure 3A1–A3). The small intestines of the infected non-treated rats showed normal histological structures with well-formed excystation and the appearance of trophozoite *Giardia lamblia* parasites (Figure 3B1–B3). Histological sections of the small intestines of the infected rats treated with quercetin showed mild inflammatory infiltrate limited to the mucosa, with the non-appearance of either the diagnostic or infective Giardia stages and some lymphocytes present in the mucosa lining (Figure 3C1–C3). Histological sections of the intestines of infected rats treated with chitosan showed degenerated cysted stages of *Giardia lamblia* (Figure 3D1–D3). Histological sections of the intestines of the infected rats treated concurrently with the combination of quercetin and chitosan afforded high recovery rates of the normal intestinal internal structures with normal myenteric ganglions (Figure 3E1–E3).

### 3.3. Transmission Electron Microscope (TEM) Examinations

**Figure 4:**
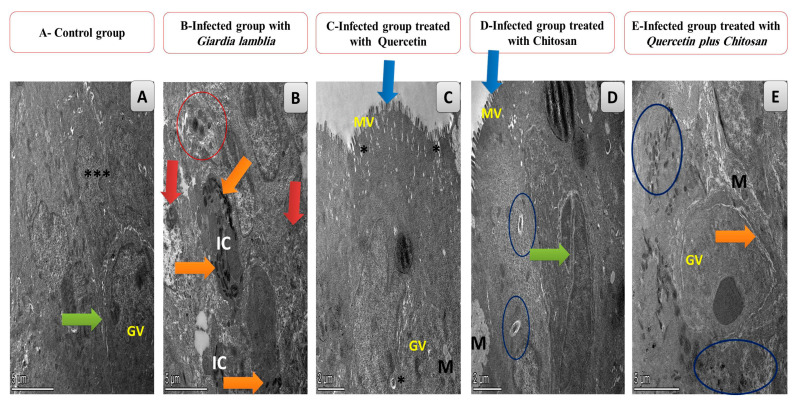
TEM images of tissue sections of the small intestines. (**A**) control group, showing normal, uniform patterns of regular intestinal muscles (black asterisks), nuclei, and the normal appearance of enteric ganglions (green arrow) with normal lucent Golgi vesicles (GV); scale bar = 5 µm. (**B**) Infected non-treated group showing the degenerative changes in the enteric ganglion (orange arrow) along with a loss of the regularity of the intestinal muscle layers and the appearance of morphological changes (red arrow) and eosinophilic exudates due to infection (red circle) with enlarged inflammatory cells (IC); scale bar = 5 µm. (**C**) The infected group with *G. lamblia* and treated with quercetin (QC) showing the normal appearance of intestinal muscles with microvilli (blue arrow) (MV) and ruminants of *Giardia lamblia* cysts (black asterisk) with mid-sized mitochondria (M) with the appearance of normal lucent Golgi vesicles (GV); scale bar = 2 µm. (**D**) the infected group with *G. lamblia* was treated with chitosan (CH), showing the normal appearance of the intestinal muscles with microvilli (blue arrow) (MV), a normal enteric ganglion (green arrow), and mild-sized cysts of *Giardia lamblia* (blue circles) with the swelling of the mitochondria (M); scale bar = 2 µm. (**E**) Infected group with *G. lamblia* and treated with both treatments of quercetin (QC) and chitosan (CH), showing the restoration of uniform patterns of the intestinal muscles and clear nuclei and enteric ganglions of the intestinal muscles (orange arrow), with only eosinophilic exudates and inflammatory cells (dark blue circles) with the appearance of normal lucent Golgi vesicles (GV) with normal-sized mitochondria (M); scale bar = 5 µm.

TEM images of tissue sections were taken from the small intestines. The control group showed normal, uniform patterns of regular intestinal muscles with the appearance of normal lucent Golgi vesicles (scale bar = 5 µm) (Figure 4A). The non-treated infected group showed degenerative changes in the enteric ganglion, along with a loss of regularity in the layers of the intestinal muscle and the appearance of enlarged inflammatory cells (scale bar = 5 µm) (Figure 4B). The infected group treated with quercetin (QC) exhibited intestinal muscles with a normal appearance, with microvilli and degenerated *Giardia lamblia* cysts with mid-sized mitochondria (scale bar = 2 µm) (Figure 4C). The infected group treated with chitosan (CH) had intestinal muscles with a normal appearance, with microvilli, normal enteric ganglions, and mild cysts of *Giardia lamblia* with the swelling of the mitochondria (scale bar = 2 µm (Figure 4D)). The infected group treated with a combination of quercetin (QC) and chitosan (CH) exhibited the restoration of uniform patterns of the intestinal muscles, with clear nuclei and enteric ganglions of the intestinal muscles; they exhibited only remnants of the phases of *Giardia lamblia* and inflammatory cells (scale bar = 2 µm (Figure 4E)).

### 3.4. Biochemical Evaluation of Hepatorenal Functions

The current results reveal that infection with *G. lamblia* led to an elevation in liver enzymes (ALT and AST). The treatment of the infected groups with QC and/or CH demonstrated a significant decrease in the liver enzymes. The best results were found in the infected group treated with a combination of QC and CH (Table 2). The same result was recorded for kidney function, both for urea and creatinine levels; thus, promising therapeutic effects were obtained in the infected group that was treated with a combination of QC and CH.

### 3.5. Changes in Oxidative Stress in the Infected Non-Treated Group and Different Infected Treated Groups

The infected non-treated group exhibited a marked elevation in MDA levels with a significant and marked reduction in the antioxidant enzymes (SOD, CAT, and GSH). The treatment of the infected groups with QC and/or CH afforded a marked decline in the lipid peroxidation final marker (MDA), which means that there was a decline in the oxidative stress levels and a significant elevation of antioxidant enzymes (SOD, CAT, and GSH). As shown and recorded in Table 3, the results showed the high elevation and ameiolration of all the antioxidant enzymes in the groups treated with either QC and/or CH, and the group’s best elevation and recovery rate was noted with a combination of QC and CH. A combined treatment group with both QC and CH was the best ameliorative treatment, and the therapeutic treatment current results showed a decline in the oxidative stress markers.

## 4. Discussion

Giardiasis is a key cause of malabsorption and weight loss worldwide; in some cases, it may cause developmental issues in children and infants [29,30]. It may lead to an increase in resistance to different diseases, thus leading to mortality [1]. There is a need for new alternative active therapies for use against giardiasis due to the side effects of regular antiparastic drugs and the mutagenic and carcinogenic effects of some of these medications; additionally, resistance to therapeutic agents may lead to the creation of new harmful infections.

The number of giardiasis cases is constantly increasing, and millions of people are infected during their daily lives and in public places; the number of cases is expected to rise in the coming years. Therefore, great efforts with different therapies must be made to develop novel alternative therapeutic agents that are more efficient against *G. lamblia* and have reduced side effects.

There are alternative active compounds that are mostly safe and have succeeded in therapeutic contexts, especially against parasites, as reported previously [31,32].

This research trend is endorsed by the WHO as the new and recommended approach for eliminating the effects of parasites using vital medicinal, herbal, and active compounds while minimizing side effects. It is especially crucial in the fight against giardiasis due to its widespread occurrence and the appearance of strains that are resistant to routinely administered antiparastic medications [33].

A previous study [34] demonstrated that QC, either alone or when loaded with nanoparticles, exhibits anti-plasmodium activity with reduced cytotoxic effects; it also has potent antioxidant and anti-inflammatory activities. These results confirm the basis of the present study and prove that QC exhibits an anti-parasitic effect against *G. lamblia*.

The results of the current study are in complete agreement with the previous study [35], who proved the efficacy of chitosan nanoparticles (a form of chitosan with increased bioavailability) when used in the treatment of giardiasis. This treatment was proven safe in vivo and in vitro, and the researchers considered nano-chitosan to be a good candidate for the treatment of giardiasis. The combination of QC and CH in the current study improved the bioavailability of CH, improving its efficacy as an antiparasitic therapeutic combination and confirming its safety for use on hepatic tissues.

Another recent study [34] demonstrated that using three different concentrations of QC, either alone or loaded on phytosome, produced an anti-malarial effect with no cytotoxicity. The authors noticed that the use of QC showed surprising effectiveness in vivo and in vitro 72 h after treatment; these current findings explain the observed results obtained in the present study and prove the efficacy of treating infected groups with QC and may explain the high level of efficacy of a combination of both QC and CH.

The current study revealed that CH was an effective agent against giardia and this effect was noticed to be elevated in case of combination with QC treatment, the current findings proved the previous concept of [35] who reported that CH act as an antimicrobial agent via it’s potent interaction with the cellular surfaces molecules. Additionally, it may explain that CH can open tight junctions of the cell surface, due to its high mucoadhesion characteristic and rapidly changing pH that elevate its solubility and bioavailability [36]. This explains the current observed results and notice of amelioration afforded by using CH and elevation of this effect with a synergistic effect when combined with QC.

Chitosan (CH) could hinder the growth of microorganisms rather than killing them as previously reported by [37] but the synergistic effect between QC and CH add an extra layer of strength to their anti-parasitic effect.

Regarding the validation of the concept examined here, previous studies [38,39] confirmed that there is an urgent need to use active and natural compounds because antiparasitic drugs exacerbate parasitic resistance. Thus, this study again confirmed the current key concept by finding alternative therapeutics for use against giardiasis.

One of the most important studies was performed in Africa [40], who investigated 36 plant species that are effective against giardiasis. These studies are of great importance as there is scarce information regarding alternative therapeutics against giardiasis, and more prospective studies may lead to the identification of the best alternative natural or active compounds.

A previous study [41] proved that QC exhibited a hepatoprotective effect, in addition to its antioxidant activity, due to the elevation of antioxidant enzymes and a reduction in MDA; these findings are in current agreement with the results obtained in the present study. It is known that the liver plays an important role in the metabolism and detoxification of various drugs [41]. QC was reported to alleviate liver steatosis induced by ethanol in ethanol-fed mice [42]. Thus, QC was shown to play an important role in the amelioration of hepatocellular functions and the improvement of lipophagy.

Similarly to the present study, the research conducted previously [43] showed that QC reduced and inhibited cellular growth in colon cancer. This provides additional confirmation of the efficacy of QC regarding the intestinal mucosa and its protection as shown in the histological and ultrastructural sections with almost normal intestinal structures in the groups that were treated with either QC or CH and the high effect was recorded in the combined group treated with both QC and CH.

As reported previously, QC is considered an excellent antioxidant agent and a potent scavenger of free radicals due to its pharmacophores [44]. This may explain the vital role of antioxidants as a new line in the therapeutic protocols against *G. lamblia* due to its ability to scavenge these free radicals, which cause severe damage to the intestinal tissues and thus reduce its physiological potency against parasites, using agents with high antioxidant activities either natural or active compounds will lead to better treatment and a worthy point of investigation due to their antiparasitic effect with fewer side effects.

Confirming this concept, there is a potent role of antioxidants against parasitic infections and a relation between high oxidative injury and high parasitic infection, a previous study [45], found that MDA levels for patients infected with giardia were significantly higher than that in the control group. This confirms the current observed results that investigated the potent synergistic effect of QC and CH against parasitic infection induced by *G. lamblia* and the reduction of oxidative injury due to its role in parasitic infections [46].

Regarding the histological analysis that showed the potent antiparasitic effects of both QC and CH on intestinal sections as compared to the intestine of the infected non-treated group that exhibited higher and large numbers of *G. lamblia* trophozoites, marked atrophy in the structure of the villi, and marked lymphocyte infiltration, all these findings are consistent with the previous recorded studies [47,48]. As observed in the intestinal sections of the infected non-treated group that showed damage to the intestinal mucosa in giardiasis and involves significant alterations in the microvilli and dysfunction of the intestinal epithelial barriers [49], Controversely, groups treated with either QC or CH or their combination that showed great ameliorative anti-parasitic effects with the restoration of damaged intestinal tissues.

The effectiveness of QC or its nanoformulations against various kinds of oxidative damage may solve the problems of antiparasitic drug resistance. CH was used to design a delivery system for QC to better release the QC in its active form, as demonstrated in a study that used a combination of QC and CH containing folic acid, which significantly inhibited breast cancer cells [50].

Thus, the formula of QC–CH displays effective gastrointestinal stimulation and enhances cellular uptake, thus reducing the effects of giardiasis and alleviating other side effects.

Finally, the parasitological, histological, ultrastructural, and biochemical investigations demonstrated the significant synergistic improvement afforded when using both QC and CH against the infection induced by *G. lamblia*.

## 5. Conclusions

In conclusion, the current study revealed that QC, in combination with CH, exhibits a promising therapeutic effect against giardiasis in infected male albino rats. A combination of both QC and CH holds promise as an antioxidant, hepatoprotective, and antiparasitic alternative therapy for giardiasis. More prospective studies are needed to observe the mechanism of action and the antitoxic effects against giardia with different dosages.

## Figures and Tables

**Table 1:** Fecal cysts in the stool of the studied infected treated and non-treated groups.

Fecal Cyst Count	Infected Non-Treated Group	Infected Group Plus QC	Infected Group Plus CH	Infected Group Plus QC and CH
Range of cysts	9.9–14.65	1.05–1.6 **	1.4–2.8 **	0.1–0.7 ***
% of reduction	0%	90%	85%	99%

***: High significant (*p* < 0.001) **: Significant (*p* < 0.05).

**Table 2:** Effect of treatment of the infected non-treated group and the infected groups and treated with QC and CH either alone/or their combination on liver and kidney functions expressed as (mean ± S.E).

	Control Group	Infected Non-Treated Group	Infected Group + QC	Infected Group + CH	Infected Group + QC + CH
ALT (U/L)	15.25 ± 2.05 ^e^	65.25 ± 3.25 ^a^	40.02 ± 4.25 ^bc^	47.09 ± 3.25 ^b^	24.69 ± 2.69 ^d^
AST (U/L)	18.25 ± 2.87 ^e^	77.58 ± 3.25 ^a^	35.69 ± 2.98 ^c^	38.98 ± 4.56 ^bc^	22.68 ± 1.68 ^d^
Creatinine (mg/dL)	0.63 ± 0.12 ^e^	1.08 ± 0.41 ^a^	0.70 ± 0.32 ^c^	0.79 ± 0.14 ^bc^	0.66 ± 0.41 ^de^
Urea (mg/dL)	24.02 ± 2.67 ^e^	32.02 ± 5.02 ^a^	27.05 ± 3.02 ^c^	28.05 ± 2.54 ^bc^	24.69 ± 3.65 ^de^

Means within the same column in each category carrying different letters are considered significant level at (*p* ≤ 0.05), the highest mean value has a symbol (a), and decreasing values were assigned alphabetically.

**Table 3:** Changes in oxidative/antioxidant parameters of the antioxidant enzymes in the liver tissues of the infected control non-treated group and the infected groups treated with either QC, CH, and their combination (mean ± S.E).

	Control Group	Infected Non-Treated Group	Infected Group + QC	Infected Group + CH	Infected Group + QC + CH
SOD (U/g)	18.68 ± 2.02 ^ab^	10.03 ± 1.65 ^e^	13.02 ± 1.87 ^cd^	12.87 ± 1.98 ^d^	16.25 ± 2.32 ^b^
CAT (U/g)	8.65 ± 1.87 ^ab^	1.65 ± 0.98 ^e^	6.35 ± 1.25 ^cd^	5.98 ± 1.68 ^d^	8.05 ± 2.69 ^b^
GSH (U/g)	14.25 ± 2.65 ^a^	5.25 ± 1.65 ^d^	10.96 ± 2.98 ^c^	10.09 ± 1.87 ^c^	12.98 ± 3.65 ^b^
MDA (U/g)	10.09 ± 1.69 ^e^	18.69 ± 1.65 ^a^	13.75 ± 2.36 ^c^	14.52 ± 1.65 ^bc^	12.02 ± 1.65 ^d^

Means within the same column in each category carrying different letters are considered significant level at (*p* ≤ 0.05), the highest mean value has a symbol (a), and decreasing values were assigned alphabetically.

## Data Availability

The data presented in this study are all available in this article.
